# Evaluation of Bax and Bak Gene Mutations and Expression in Breast Cancer

**DOI:** 10.1155/2014/249372

**Published:** 2014-02-09

**Authors:** Naglaa Mohamed Kholoussi, Sobhy E. H. El-Nabi, Nora Nassef Esmaiel, Naser Mohamed Abd El-Bary, Ahmed F. El-Kased

**Affiliations:** ^1^Immunogenetics, National Research Centre, Cairo 11516, Egypt; ^2^Faculty of Science, Minufiya University, Menufia, Egypt; ^3^Molecular Genetics and Enzymology, National Research Centre, Cairo 11516, Egypt; ^4^Faculty of Medicine, Minufiya University, Menufia, Egypt

## Abstract

Genetic analyses have provided evidence to suggest that Bax and Bak are the essential genes for apoptosis in mammalians cells. This study aimed to search for biomarkers in breast cancer to be used as prognostic markers for the disease. The Bak and Bax genes expressions were analyzed in 23 breast cancer patients by RT-PCR technique. SSCP technique was used to detect the mobility of the abnormal fragment in Bak exon 4. PCR for Bax promoter was digested with Tau 1 restriction enzyme to identify a single polymorphism G(-248)A. The expression of Bak gene is related to several clinical factors of breast cancer. The analysis of Bax RNA showed 4 isoforms of Bax with different distributions in the normal and tumor tissues. These isoforms were Bax **α**, d, **δ**, and **ζ**. Exon 4 had a normal pattern in all cases of breast cancer. There was a statistically significant difference in the frequency distribution of the G(-248)A genotypes in the breast cancer tissues with grade 3+high, T2 stage, lobular +other, and PR −ve subgroups. In this study, Bak expression seems to lead to development of breast cancer and affects the disease progression. Also, Bax d and Bax **δ** could be used as risk factor and biomarker for breast cancer with the distribution of G284A.

## 1. Introduction

Worldwide, breast cancer is the fifth most common cause of cancer deaths (after lung cancer, stomach cancer, liver cancer, and colon cancer) [1]. According to the Egyptian national cancer institution, the leading cancers in Egyptian patients are the urinary bladder (32.67%), gastrointestinal tract (22.24%), breast (13.15%), and lymphoma (9.8%). *Apoptosis* is the process of programmed cell death that may occur in multicellular organisms [2]. Apoptosis is of major clinical relevance because deregulated apoptosis caused by the suppression, overexpression, or mutation of key apoptotic regulators contributes significantly to numerous human diseases [3]. Bax and Bak are the gateway to the mitochondrial pathway of apoptosis. Cells that are doubly deficient in the two multidomain proapoptotic Bax and Bak fail to release cytochrome c and are resistant to all apoptotic stimuli that activate the intrinsic pathway [4, 5].

Activation of Bax and Bak during apoptosis involves multiple conformational changes that are accompanied by their mitochondrial intramembranous homooligomerization [6]. The Bax gene encodes several variants of Bax, the principal form is Bax *α* [7]. The Bax gene can produce different proteins through alternative mRNA splicing mechanisms, including *α*, *β*, *γ*, and *δ* isoforms of Bax [8]. The functions of some of these variant proteins may be different from the most abundant forms of Bax p21-Bax-a. Exon 4 encodes the BH3 region. The membrane anchoring region has also been shown to be important for the apoptotic activity of Bak. In studies using truncated Bak molecules, it has been reported that the truncated molecule, which includes BH3 but not the membrane anchoring region, retains Bcl-xL binding capacity but exhibits a reduced cell killing function due to altered subcellular localization [9]. Gene transfer mediated elevations in BAK protein levels accelerate apoptosis induced by growth factor deprivation in murine lymphoid, lung cancer, and breast cancer cells. Bak functions primarily as a promoter of apoptosis [10]. Saxena et al. [11], mention that, they found a single-nucleotide polymorphism (SNP), a G to A transition, 125 nucleotides upstream from the start of transcription, and 248 nucleotides from the start of translation of Bax. This SNP was associated with reduced protein expression, higher stage of the disease, and failure to achieve complete response to treatment in patients with CLL. Mutations in the promoter and coding regions of the Bax gene have been shown to affect protein expression and function in many cancers [12].

## 2. Material and Method

### 2.1. Patients and Samples

Normal and tumor tissues were obtained at the time of surgical resection from 23 subjects with breast cancer. Clinical information obtained from the patients medical records; the main age of them was 52.7 ± 11.7. The subjects include 23 normal tissues (fibroadenosis) and 22 tumor tissues. All possible surgical specimens of breast cancer were collected on liquid nitrogen N2 and subjected to isolation of RNA and DNA. The study protocol was approved by the medical research ethics committee of National Research Center.

### 2.2. Reverse Transcription-PCR

Total RNA was obtained using Biozol reagent (bioflux) Biozol-RNA Kit and quantified by spectrophotometry, and cDNA was prepared using RevertAid first strand cDNA synthesis Kit (Fermentas) at 42°C. PCR amplification of Bak expression and Bax isoforms were done from 100 ng cDNA using the primers: Bak F 3′ACGCTATGACTCAGAGTTCC5′, Bak R 3′CTTCGTACCACAAACTGGCC5′ Bax F 5′ATGGACGGGTCCGGGGAGCA3′, and Bax R 5′CCCAGTTGAAGTTGCCGTCA3′. As control, amplification of the housekeeping gene GPDH was done. The products were electrophoresis on 2% agarose gels stained with ethidium bromide. The expression level of Bak gene was quantified by gel documentation and normalized against the internal control GAPDH expression [13, 14]. The isoforms of Bax were detected by their molecular size. Kapelan Bio-Imaging solutions software was used to measure the Bak expression and the length of the Bax isoforms, version 2.7.2 (http://www.LabImage.com/) 1999–2005, Germany.

### 2.3. DNA Extraction

Normal and tumor tissues of 43 subjects (23 normal and 22 tumors) were collected on liquid N2. DNA was extracted by salting out technique as described by [15].

### 2.4. *Bax* 5_-Untranslated (Promoter) Region G(-248)A Polymerase Chain Reaction Analysis

A 280-base pair fragment of the *Bax* 5_-untranslated (promoter) region was amplified using the following primer pair: 5_TTAGAGACAAGCCTGGGCGT-3_ and 5_CAATGAGCATCTCCCGATAA-3_. The polymerase chain reaction (PCR) mix contained 10x PCR reaction buffer, 10x dNTPs, and 200 Ng for each primer. After initial denaturation at 95°C for 5 minutes, 35 cycles of amplification were performed, consisting of denaturation at 95°C for 30 seconds, annealing at 48°C for 30 seconds, and extension at 72°C for one minute, with a final extension step of 72°C for 7 minutes. A 12 *μ*L aliquot of each PCR product was digested with 1 unit of Tau1 restriction enzyme (MBI Fermentas, Helena Biosciences, Sunderland, UK) at 55°C for 15 minute. The Tau1 enzyme recognizes the sequence GCSGC, and the presence of the A allele results in the sequence ACSGC, thus abolishing the restriction site. The digested products were electrophoresed on a 2% agarose gel, stained with ethidium bromide, and visualized using ultraviolet. The Tau 1 restriction enzyme digest produced three possible band patterns. The normal allele was completely digested to give two bands, 234 bp and 46 bp; the mutant allele was uncut and gave a single band of 280 bp, and all three bands were present in patients showing heterozygosity of the G(-248)A polymorphism.

### 2.5. Detection of Mutation in Bak Exon 4

Exon 4 of Bak was amplified from genomic DNA via polymerase chain reactions in 35 *μ*L reaction mix containing 2.5 u/*μ*L of Taq polymerase (DyNAzyme II DNA polymerase), 10x PCR reaction buffer, 10x dNTPs, and 200 Ng for each primer Bak exon 4 F 5GGCAGGGTATGGTATGGTTG3 and Bak exon 4 R 5TCCCGACTGCCTGGTTACTG3. After initial denaturation at 95°C for 5 minutes, 35 cycles of amplification were performed, consisting of denaturation at 95°C for 1 minute, annealing at 55°C for 1 minute, and extension at 72°C for 1 minute, with a final extension step of 72°C for 7 minutes. The mobility of the abnormal fragment of exon 4 was detected by SSCP technique where 10 *μ*L of each PCR product was denaturated at 95°C for 10 minutes and electrophoresis for 4 hours at 300 v in 10% polyacrylamide gel. After electrophoresis, the gel was stained with a 0.5 mg/mL solution of ethidium bromide for 20 minutes and then washed with water for five minutes. Ethidium bromide stained bands were visualized using a 340 nm ultraviolet viewing box [16].

### 2.6. Statistical Analysis

The statistical analysis of the results was done by the program of SPSS (Statistical Package for Social Science), copyright of Ecosoft Inc. V..S (1995). The analysis was performed using two-tail *t*-test, ANOVA (one-way) analysis, and chi square. Significant level was accepted at *P* ≤ 0.05. The subjects are divided by several ways according to three different criteria: divided according to types of tissues to normal (N) and tumor tissues (N), divided according to the clinical factors to give four groups, and divided according to the hormones receptors status.

## 3. Results

### 3.1. Bak Had Higher Expression in the Tumor Tissues Than Normal Ones

**Figure 1:**
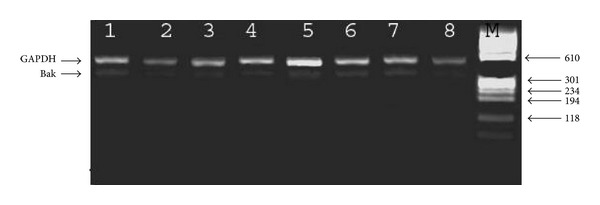
Agarose gel electrophoresis shows expression of Bak mRNAs in breast cancer and normal tissues by RT-PCR analysis. GAPDH is an internal control with 469 bp, Bak mRNA expression with 364 bp. Lanes 1–5 and 7 are breast cancer tissues and lanes 6 and 8 are normal tissues.

**Figure 2:**
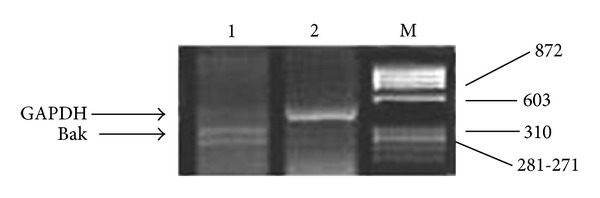
Agarose gel electrophoresis shows Expression of Bak mRNAs in breast cancer and normal tissues by RT-PCR analysis. GAPDH is an internal control with 469 bp, Bak mRNA expression with 364 bp. Lane 1 shows a mutant pattern of Bak mRNA. Lane 2 is breast cancer tissue. M is ΦX–174 Hae III markers.

To explore the possible involvement of Bak in breast cancer with reference to clinical factors, the representative pattern of the Bak expression, measured by RT-PCR, is shown in Figures 1 and 2. The expression levels were quantified by gel documentation program and normalized against the internal control GAPDH expression. First, the mean of the Bak expression in normal tissues was 0.350. The mean of Bak expression in tumor tissues >0.350 was significantly higher than that of tumor tissues <0.350 and normal tissues (*P* = 0.001) (Table 1). *In single locus analysis*, the tumor tissues >0.350 had a significant high Bak expression (0.019).

The expression levels of Bak gene may be related to several clinical factors of breast cancer as shown in Table 1. There was not a statistically significant in Bak mean expression in each group of the clinical factors. Also in case of hormone receptors status groups, there was not a significant difference in the Bak expression mean.

*In single locus analysis*, the mean of Bak expression was significantly higher in tissues with grade 3 and higher than normal tissues (*P* = 0.006) as shown in Table 1. Also, the mean expressions was significantly higher in node 2 (*P* = 0.02) than in the normal tissues. In stage 4, ER positive and PR negative expressions of Bak were high but with nonsignificant (*P* = 0.1). There was no significant correlation between Bak expression and invasive carcinoma.

Interestingly, one different pattern of Bak was appearing as double strand, Figure 2, and also one case has no Bak expression at all and remarkably the two cases were T3 stage.

### 3.2. Bax Gene Expression Shows That Bax d and Bax Δ Had a Significant Association in the Tumor Tissues Than the Other Isoforms

**Figure 3:**
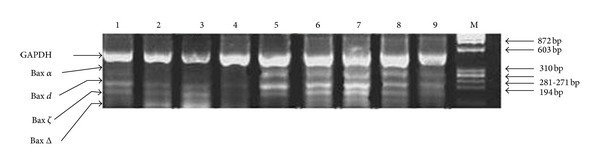
Agarose gel electrophoresis shows RT-PCR analysis using Bax mRNA to amplified Bax isoforms. GAPDH is an internal control with 469 bp, Bax *α* with 322 bp, Bax d with 270 bp, Bax Δ with 175 bp, and Bax *ζ* with 132 bp. Lanes 1–9 are breast cancer tissues. M is ΦX-174 Hae III markers.

The expression of Bax was analyzed by RT-PCR of mRNA in normal tissues and tumor tissues. Four bands were apparent with molecular sizes, 322 bp, 270 bp, 175 bp, and 132 bp. When these lengths were applied to NBCI and GeneLoc databases, found that these bands are Bax *α* (NCBI Reference Sequence: NM_138761.3), Bax d (GenBank: AW008643.1), Bax Δ (NCBI Reference Sequence: NM_138763.3), and Bax *ζ* (GenBank: AF250190.1), Figure 3.

Bax *α*, Δ, and *ζ* were documented in cancer cells but variant d is documented in thymus and pooled tissues (NCBI, ACEView). This variant was the first to document in breast cancer. When compared with *α* sequence Bax d was an alternative truncated variant at c terminal. *In single locus analysis*, there was a significant presence of Bax d in tumor tissues (*P* = 0.01) as shown in Table 2.

The relation between isoforms and clinical factors of breast cancer was analyzed as shown in Table 2. Bax d has a high presence in grade 2 (*P* = 0.08) except that there was not a significant appearance of Bax isoforms in each subgroup.

*In a single locus analysis*, Bax Δ, opposite to other isoforms, has a strongly significant presence in grade 2 (*P* = 0.007). Also, in tumor stages, the Bax d and Bax *δ* have a significant appearance in T2 (*P* = 0.003). Interestingly, Bax *α* has no expression in T3. In case of invasive duct carcinoma, there was not a significant appearance of Bax Δ (*P* = 0.09). Bax *ζ* has a low appearance in the tumor tissues and in all the clinical factors. No significant presence was found in node, ER or PR factors as shown in Table 2.

Two cases only had no Bax expression at all and there was not a similarity between them except that they were a duct carcinoma.

### 3.3. Bak Exon 4 Had a Normal Pattern in All Breast Cancer Cases

**Figure 4:**
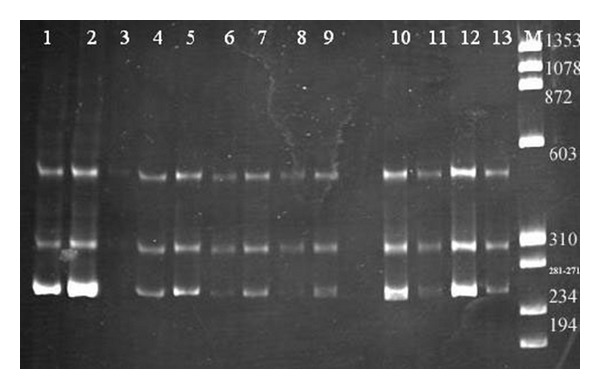
SSCP analysis of PCR amplified products of Bak exon 4. Electrophoresis on 10% polyacrylamide gel at 4°C shows normal patterns. Lanes 1, 2, 3, 4, 6, 7, 9, 12, and 14 represent SSCP analysis of exon 4 in breast cancer tissues. Lanes 5, 8, 11, and 13 represent normal tissues. M is ΦX-174 Hae III marker.

Exon 4 encodes the BH3 region in Bak gene. It was amplified by PCR and followed by SSCP to detect mobility of the abnormal fragment but in all cases (35 specimens) exon 4 had a normal pattern, Figure 4.

**Figure 5:**
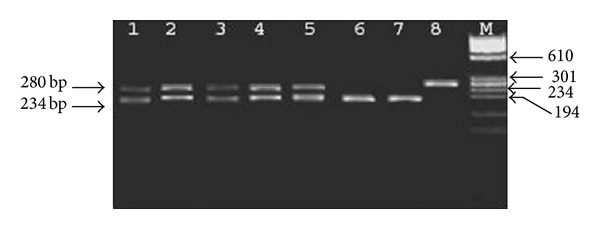
Restriction enzyme assay by Tau 1 enzyme for Bax promoter SNP electrophoresis on 3% agarose gel. Lanes 1–5 were heterozygote SNP (G-A), Lanes 6 and 7 were normal homozygote (A-A), and lane 8 was mutant homozygote (A-A). M was ΦX-174 Hae III markers (M).

*Bax promoter G-248A SNP had a significant presence in the breast cancer tissue*, PCR for Bax promoter was digested with Tau 1 restriction enzyme to identify a single polymorphism G(-248)A. The digestion gave three patterns: the wild types normal (G-G) 234 bp, and 46 bp bands; wild type/variant (G-A) 280 bp, 234 bp, and 46 bp bands; and the variant (A-A) 280 bp band only as shown in Figure 5. The distribution of this SNP among the breast cancer tissues and normal tissues is shown in Table 3. There was not a significant presence of the three genotypes between the normal and tumor tissues. *In the single locus analysis*, in normal group there was a significant statistical presence of the G-A genotype (*P* = 0.01 Bax G-A). When the clinical factors subgroups were used as frequency, there was a statistical significant presence of the G-A genotype in grade 3+high (*P* = 0.002), T2 stage (*P* = 0.009), and lobular +other (*P* = 0.01) as shown in Table 3. Also PR –ve subgroups show a significant presence of G-A genotype (*P* = 0.03), Table 3.

*In further stratification analysis*, variant genotypes were combined (i.e., GA+AA) to see the incidence of the SNP in the tumor tissue and its effect. In L.N. subgroup there was a significant distribution of the combined variant frequency (*P* = 0.04), Table 3. *In the single locus analysis*, when the clinical factors were used as frequency, there was a significant presence of combined variant (G-A+A-A) in normal tissues (*P* = 0.003), and in tumor tissues (*P* = 0.001), Table 3. Also, grade 2 and grade 3+high show a significant presence of combined variant (*P* = 0.004 and 0.002). Moreover, the breast cancer with T2 stage shows a significant presence of combined variant (*P* = 0.03). Interestingly, in case of N0 there was not a presence of wild type (*P* = 0.000). ER +ve tumor tissue has a significant association of combined variant (*P* = 0.003). In both PR subgroups (i.e., –ve and +ve), they have a significant association of the combined variant.

## 4. Discussion

*In the present study*, mRNA expression of proapoptotic Bak has been increased in the tumor tissues than in the normal tissues. Moreover, mRNA level of Bak was increased more significantly in N3 cases than the other L.N. stages. Koda et al. [17] indicated that the changes in the expression of Bak coincide with breast cancer development and progression.

Eguchi et al. [13] indicated that Bak expression shows a significant increase at high tumor stages (T3 and T4). The same results had been found where Bak mRNA level shows a significant increase in the T3 and T4 tumor stages.

Zohny and El-Shinawi [18] reported that Bak mRNA was markedly decreased in bone metastatic patients compared to nonmetastatic patients; however, significant expression of Bak mRNA was observed in bone metastatic cases compared to benign patients. In contrast, in the present study, N2 shows a significant increase in Bak mRNA opposite to N0 or N1. Moreover, in the present study, invasive duct carcinoma shows a high Bak expression. But Koda et al. [17] and Eguchi et al. [13] reported no statistically significant differences in Bak expression in invasive cancer.

Ghayad et al. [19] and Eguchi et al. [13] reported that the ER-positive breast cancer cells have a significantly overexpressed Bak mRNA. Sorbello et al. [20] also said that, Qiang-Song et al. [21] reported that deficiency of Bak expression is closely correlated with occurrence and development of tumors. It was also indicated that Bak overexpression might arrest the cell cycle and induce apoptosis in cancer cells. Graber [22] explained the increase of Bak expression in tumor tissues where the tumor cells and the stroma cells expressed only low levels of Bak. In contrast, in regions adjacent to the tumor, which showed chronic inflammation, there was always high expression. Taken together, these results suggest that in pancreatic cancer Bak expression and programmed cell death are present in cells that are localized in regions of chronic inflammation surrounding the pancreatic cancer cells but not in the tumor cells themselves, a situation that may facilitate tumor growth and spread.

This may explain the results here, where the Bak expression was higher in tumor tissues than in normal tissues. Also it explained why the Bak expression was higher in the N3, N4, and high tumor stages than in N0. Another explanation to the increasement of Bak is that the apoptosis is a cascade pathway in which many factors participate in it, so Bak may not be the reason or the factor which causes the tumorigenesis here.

Eguchi et al. [13] said that no mutations have been reported so far but here we have two mutations and interestingly the two have the same clinical features which were T3, grade 2, ER+, invasive duct carcinoma, and LN1. This suggested that Bak mutation may be a biomarker for these features.

Alternative splicing is the process by which splice sites in the precursor mRNA transcripts are differentially selected to generate structurally and functionally distinct mRNA and protein isoforms from individual genes [23]. Akgul et al. [8] reported that the mRNA expression profile has underscored the complexity of the alternatively splicing patterns of the Bax gene. *In the present study*, RT-PCR had shown four Bax isoforms *α*, d, Δ, and *ζ*. Interestingly, Bax d and Bax Δ had a much significant association in the tumor tissues than the other isoforms, which suggest that Bax d and Bax Δ may association with tumor progress and development. Maia et al. [24] reported that BAX-*α* was detected in all cases tested, constituting the prevalent form. BAX-Δ, which lacks the death domain encoding exon 3, was detected in all patients tested. Also, most of patients expressed BAX-*σ* and BAX-*ζ*. But in the present study, the variants d and Δ were the most presented isoforms in the tumor tissues while *α* has a low presence and *ζ* has the lowest presence.

Bax *α* is activated during apoptosis as a consequence of changes of its conformation, which is believed to be mainly regulated by the intramolecular interaction between its N- and C terminal regions [7, 25]. In BAX-Δ, the in-frame fusion of exon 2 and exon 4 results in a molecule lacking the BH3 death domain predicted to be unable to dimerize with BCL-2 family members [24]. Also, Bax d is a truncated variant of Bax *α* from the C terminal (NCBI, ACEView). Maia et al. [24] elucidate that it has been suggested that BAX-Δ or other inactive, death domain-lacking isoforms may affect cell survival by disrupting the delicate balance between antiapoptotic and proapoptotic effectors as shown for p53 dysfunction or BCL-2 overexpression. For all that, changes in the conformation of Bax, its relocation from cytosol to mitochondria, and its oligomerization in the mitochondrial membrane are presumed to be important for the initiation of apoptosis [26–28]. Moreover, Suzuki et al. [29] said that the intramolecular contacts between the Bax C-terminus and its BH-3 domain are thought to prevent the docking of Bax at the mitochondrial surface and to prevent the binding via BH-3 interaction with other family members such as Bcl-xL.

Bak, which is a member of the Bcl-2 family, shows the presence of the BH1, BH2, and BH3 homology domains and a membrane anchoring region. The Bak gene functions by binding and inhibiting the antiapoptotic molecule Bcl-xL, thereby inducing apoptosis. The BH3 domain of the Bak gene, encoded by exon 4, has been shown to be responsible for its ability to induce apoptosis as well as to bind Bcl-xL [30].

*In the present study*, there was evidence of aberrant band shifts on SSCP gel suggested that there was no alteration in exon 4 of Bak gene in breast cancer cells and this thesis was the first analyzed exon 4 in breast cancer. This is confirmed by the results of Kondo et al. [10] who analyzed exon 4 in 24 gastric cancer but they have no evidence of sequence alteration in this exon. Also, Yoshino et al. [31] found that there was no mutation of Bak gene in prostate cancer samples.

The BAX gene has been mapped to chromosome 19q13.3. Recently, a SNP located within the 5′-untranslated region of the BAX promoter, G-248A (rs4645878), was reported to be associated with both reduced expression of BAX and altered susceptibility to chronic lymphocytic leukemia [11, 12, 32–34].

*In this context*, G-A SNP was found significantly in the grade 3 than grade 2, lobular carcinoma than duct carcinoma. It also characterized associative with PR− than PR+. Interestingly, normal tissues had a significant association of the G-A SNP but the tumor tissues had no significant distribution of the polymorphism.

*In further stratification analysis*, when variant genotypes were combined, the novel single nucleotide polymorphism was associated with T2 stage, N0, lobular carcinoma, and ER +ve tumor tissues which may be a marker for these factors. The significant association of SNP in the tumor tissues may suggest that this SNP association with tumor progress. Saxena et al. [11], confirm these results where they said that SNP was associated with low Bax protein expression, disease progression, and a failure to achieve complete response to therapy. Chen et al. [35] said that the Bax AA variant genotype exhibited an elevated risk of SCCHN. Lahiri et al. [36] elucidate that the Bax SNP was associated with a more advanced Binet stage at diagnosis, supporting a potential role for this variant in CLL disease progression. Starczynski et al. [37] failed to find a significant frequency of the G(-248)A polymorphism in CLL cases when compared with healthy control. Neither did they find a bias in the distribution of the polymorphism among CLL cases.

*In conclusion*, the apoptosis is a multigenetic pathway, and so one of these factors may be normal and respond normally to the body signals but the other factors may cause disturbance in the apoptosis pathway and lead to cancer. In this study, Bak expression seems to lead to development of breast cancer and affects the disease progression. Also, Bax d and Bax *δ* could be used as risk factor and biomarker for breast cancer.

The G284A SNP of Bax promoter has a significant association in the breast cancer subject which may suggest that this SNP acts as a risk factor. Bak exon 4 mutation does not seem to be common in the breast cancer.

## Figures and Tables

**Table 1:** The coloration between Bak expression and clinical factors.

Factors	Bak expression			
	Number of tumors^a^	Mean expression level	P*	P**
Normal tissue	4	0.350	0.001 P***	0.077 0.019
Tumor < 0.35	5	0.201		
Tumor > 0.35	8	0.598		

Grade				
2	16	0.458	0.185	0.427
3+ high	3	0.672		0.006

T stage				
T1	3	0.355	0.306 P***	0.953
T2	7	0.462		0.084
T3	3	0.407		0.762
T4	6	0.644		0.061

L.N.				
N0	6	0.448	0.642 P***	0.45 0.520 0.026
N1	6	0.478		
N2	7	0.575		
N3	1	0.277		

Histopathology				
Invasive duct carcinoma	17	0.479	0.557	0.323
Lobular + other	3	0.576		0.157

ER				
Positive	9	0.562	0.520	0.195
Negative	6	0.464		0.2

PR				
Positive	7	0.544	0.98	0.29
Negative	7	0.547		0.1

^a^Some cases are omitted because of missing values.

P*:  Student's *t*-test used to determine the significance of the Bak expression among subgroups. Statistical significance was at *P* ≤ 0.05.

P**:  two sample *t*-test used to determine the significance of Bak expression between each subgroup and the mean of normal tissues. Statistical significance at *P* ≤ 0.05.

P***: ANOVA test used to determine the significance of Bak expression among subgroups. The statistical significance was at *P* ≤ 0.05.

**Table 2:** Statistical analysis of the Bax isoforms distribution according to clinical factors.

Factors		Bax *α*			Bax d			Bax *δ*			Bax *ζ*			P**
Number of tissues^a^		Number of tissue	%	P*	Number of tissue	%	P*	Number of tissue	%	P*	Number of tissue	%	P*
Normal tissue	4	2	50	0.71	3	75	0.6	4	100	0.3	3	75	0.2	0.4
Tumor tissue	20	12	60		17	85		16	80		8	40		0.01

Grade														
2	16	8	50	0.6	13	81.3	0.08	14	87.5	0.4	6	37.5	0.3	0.007
3+ high	3	2	66.7		1	33.3		2	66.7		2	66.7		0.7

T stage														
T1	3	2	66.7	0.25	2	66.7	0.1	2	66.7	0.2	2	66.7	0.7	1
T2	7	4	57.1		7	100		7	100		2	28.6		0.003
T3	3	—	0		1	33.3		2	66.7		1	33.3		0.4
T4	4	4	66.7		3	50		3	50		2	33.3		0.7

L.N.														
N0	6	3	50	0.84	5	83.3	0.8	6	100	0.3	3	50	0.7	0.14
N1	6	4	66.7		5	83.3		5	83.8		3	50		0.5
N2	7	4	57.1		5	71.4		5	71.4		2	28.6		0.3
N3	1	—	0		—	0		—	0		—	0		

Histopathology														
Invasive duct	17	8	47.1	0.53	12	70.6	0.3	13	76.5	0.3	7	41.2	0.7	0.09
Labular + other	3	2	66.7		3	100		3	100		1	33.3		0.18

ER														
Positive	9	5	55.6	0.8	6	66.7	0.5	7	77.8	0.6	3	33.3	1	0.3
Negative	6	3	50		3	50		4	66.7		2	33.3		0.7

PR														
Positive	7	3	42.9	0.6	5	71.4	0.6	5	71.4	1	2	28.6	0.5	0.7
Negative	7	4	57.1		4	57.1		5	71.4		3	42.9		0.3

^a^Some cases are omitted because of missing values.

P*: significant distribution of each isoform between the same groups by chi-square test. Statistical significance was at *P* ≤ 0.05.

P**: significant distribution of the 4 isoforms in each subgroup by chi-square. Statistical significance was at *P* ≤ 0.05.

**Table 3:** Chi-square test was done for the distribution of the Bax G-248A SNP.

Factors		G→G			G→A			A→A			G→A + A→A			*P* ^1^	*P* ^3^
Number^a^		Number	%	*P*	Number	%	*P*	Number	%	*P*	Number	%	*P* ^2^	
Normal	18	5	27.8	0.9	11	61.1	0.6	3	16.7	0.4	14	77.8	0.9	0.011	0.003
Tumor	19	5	26.3		10	5.26		5	26.3		15	78.9		0.15	0.001

Grade															
2	17	7	41.2	0.16	10	58.8	0.7	5	29.4	0.4	15	88.2	0.4	0.2	0.004
3+ high	4	0	0		2	50.0		2	50		4	100		0.002	0.002

T stage															
T1	3	1	33.1	0.3	0	0	0.1	2	66.7	0.6	2	66.7	0.3	0.2	0.4
T2	8	1	12.5		6	75.0		1	12.5		7	87.5		0.009	0.03
T3	4	2	50		2	50		0	0		2	50.0		0.1	0.1
T4	7	2	28.6		3	42.9		2	28.6		5	71.4		0.8	0.1

L.N.															
N0	7	0	0	0.3	4	57.1	0.8	3	42.9	0.4	7	100	0.04	0.2	0.000
N1	6	4	66.7		2	33.3		0	0		2	33.3		0.2	1.2
N3 + N2	9	2	22.2		4	44.4		2	22.2		6	66.7		0.4	0.06

Histopathology															
Duct	19	6	31.6	0.3	7	36.8	0.04	5	26.3	0.3	12	63.2	0.2	0.3	0.7
Lab + other	3	—	0		3	100		0	0		3	100		0.01	0.01

ER															
Positive	11	3	27.3	0.6	5	45.5	0.4	3	27.3	0.6	8	72.7	0.6	0.5	0.003
Negative	6	1	16.7		4	66.7		1	16.7		5	83.3		0.1	0.2

PR															
Positive	9	2	22.2	0.7	4	44.4	0.2	3	33.3	0.38	7	77.8	0.7	0.6	0.02
Negative	7	1	14.3		5	71.4		1	14.3		6	85.7		0.03	0.008

^a^Some cases are omitted because of missing values.

*P*: chi-square test was used to determine the statistical significant distribution of each genotype between the subgroups. Statistical significance was at *P* ≤ 0.05.

*P* ^1^: chi-square test was used to determine the statistical significant distribution of the three genotypes in each subgroups. Statistical significance was at *P* ≤ 0.05.

*P* ^2^: the significant distribution of the combined variant (i.e., G-A + A-A) among each group by chi-square test. Statistical significance was at *P* ≤ 0.05.

*P* ^3^: the significant distribution of the wild type genotype and the combined variant (i.e., G-A + A-A) in each subgroup by chi-square test. Statistical significance was at *P* ≤ 0.05.
